# eTOXlab, an open source modeling framework for implementing predictive models in production environments

**DOI:** 10.1186/s13321-015-0058-6

**Published:** 2015-03-11

**Authors:** Pau Carrió, Oriol López, Ferran Sanz, Manuel Pastor

**Affiliations:** Research Programme on Biomedical Informatics (GRIB), Department of Experimental and Health Sciences, Universitat Pompeu Fabra, IMIM (Hospital del Mar Medical Research Institute), Dr. Aiguader 88, E-08003 Barcelona, Spain

**Keywords:** Modeling, Predictive models, Web services, QSAR, Open source, Confidential compounds

## Abstract

**Background:**

Computational models based in Quantitative-Structure Activity Relationship (QSAR) methodologies are widely used tools for predicting the biological properties of new compounds. In many instances, such models are used as a routine in the industry (e.g. food, cosmetic or pharmaceutical industry) for the early assessment of the biological properties of new compounds. However, most of the tools currently available for developing QSAR models are not well suited for supporting the whole QSAR model life cycle in production environments.

**Results:**

We have developed eTOXlab; an open source modeling framework designed to be used at the core of a self-contained virtual machine that can be easily deployed in production environments, providing predictions as web services. eTOXlab consists on a collection of object-oriented Python modules with methods mapping common tasks of standard modeling workflows. This framework allows building and validating QSAR models as well as predicting the properties of new compounds using either a command line interface or a graphic user interface (GUI). Simple models can be easily generated by setting a few parameters, while more complex models can be implemented by overriding pieces of the original source code. eTOXlab benefits from the object-oriented capabilities of Python for providing high flexibility: any model implemented using eTOXlab inherits the features implemented in the parent model, like common tools and services or the automatic exposure of the models as prediction web services. The particular eTOXlab architecture as a self-contained, portable prediction engine allows building models with confidential information within corporate facilities, which can be safely exported and used for prediction without disclosing the structures of the training series.

**Conclusions:**

The software presented here provides full support to the specific needs of users that want to develop, use and maintain predictive models in corporate environments. The technologies used by eTOXlab (web services, VM, object-oriented programming) provide an elegant solution to common practical issues; the system can be installed easily in heterogeneous environments and integrates well with other software. Moreover, the system provides a simple and safe solution for building models with confidential structures that can be shared without disclosing sensitive information.

**Electronic supplementary material:**

The online version of this article (doi:10.1186/s13321-015-0058-6) contains supplementary material, which is available to authorized users.

## Background

The increasing availability of series of compounds annotated with biological properties can be exploited for building Quantitative Structure-Activity Relationship (QSAR) models. Such models can be used as tools for improving our understanding of biological phenomena, by identifying the structural properties of the compounds that correlate with their biological properties. Also, mature and well validated models are amenable for assessing the biological properties of new compounds. This use is of particular interest for scientists involved in the development of new compounds in food, pharmaceutical or cosmetic industry. Typically, predictions produced by in silico models are not accepted blindly but used to raise alerts about probable safety issues, prioritize compounds or highlight the need of further experimental testing.

The steps in the development of a QSAR model (the model “life cycle”) can be summarized as follows [1]; first, the model is developed from an initial training series (building). Once the quality of the prediction is ascertained (validation), the model can be used for predicting the properties of compounds not present in the training series (prediction). After some time, the model can be improved by incorporating new compounds in the training series, thus widening the chemical space covered and the model applicability domain. Typically this required to re-build the model (re-training) obtaining a new version. It is always convenient to keep record of all the model versions, in cases where we need to reproduce historic predictions (“forensic” studies) or compare the quality of different model versions.

It is important to emphasize that not all QSAR models are developed for being used in routine prediction and therefore not all of them are suitable for being used in production environments. Such industrial-grade models must have certain good characteristics related with the quality of the model itself (like robustness of the predictions and a wide applicability domain) as well as some others related with the software implementation of the model. Here we will focus our attention on the requirements of the software supporting this use of the models rather than on the models themselves. Essential requirements of this software are:

- Easily installable at the production environments, typically corporate computational facilities.
- Specific support for all the steps of the QSAR model life cycle: building, validation, prediction and re-training.
- Regarding prediction; the software must guarantee that the structures of the compounds being predicted are submitted to exactly the same protocol used for the training series.
- Regarding re-training; models must be easily re-built by adding new compounds to the original training series.
- All model versions must be stored and accessible, allowing the reproduction of historical predictions.
- Predictions must be accompanied by indexes that indicate their reliability.

At present, few software offer an integrated solution for all the task involved in the QSAR life cycle [2]. Most modeling software is focused on the model development [3,4]. On-line tools like OCHEM [5] offer an interesting alternative, even if they are not suitable for the prediction of confidential structures. Frameworks like OpenTox [6] give unified access to data management, algorithms, modeling, validation and reporting, but focusing on chemical safety assessment and public standards. In practice, most research groups prefer to develop their own modeling workflows using generic tools like Orange [7], Weka [8] or KNIME [9] in academic environments or PipelinePilot [10] in corporate environments. All these tools have pros and cons but a detailed analysis shown that no one fulfilled the requirements for their use in the eTOX project [11], a public-private partnership project aimed to develop models able to predict *in vivo* toxicological endpoints in drug development. In this project, we needed open source software supporting the development, validation and use of predictive models using a wide variety of modeling techniques and fulfilling all requirements listed above. For this reason we developed eTOXlab, a highly flexible modeling framework that will be described in the following sections.

## Implementation

The requirements of software supporting the development and use of prediction models in production environments, already presented and discussed in the introduction, imposed very strict constraints to its design. More than a monolithic software application, we need a modeling framework where model developers can implement their models using heterogeneous methods. Furthermore, the final predictive system must be suitable for being installed in corporate computing facilities using diverse platforms (Windows, Linux, etc.) and integrate well with existing software handling the prediction results for data presentation or reporting.

For these reasons eTOXlab was developed as a collection of object-oriented Python modules, designed for being installed within a virtual machine (VM), which offers the predictions as web services.

**Figure 1:**
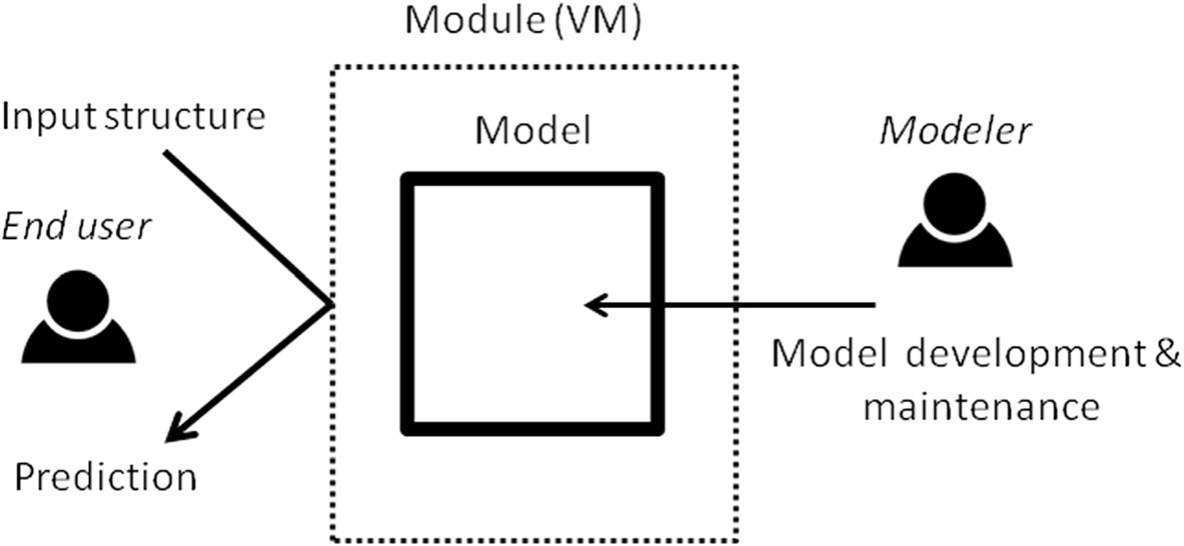
**Use of an eTOXlab model (Model).** The model is embedded in a self-contained VM, that end users can access using web services. Modelers can log in into the VM for developing new models and performing validation and maintenance.

The choice of a VM platform allows producing a self-contained prediction system that can be easily installed in academic or corporate environments or even in cloud computing infrastructures. Predictive models are exposed as web services and accessible from a web based graphic interface (Figure 1). For many end users this VM can be seen as a “black box” where they submit a molecular structure and obtain a prediction. Web services are widely used in drug design [12], since they offer easy access to data or computation resources. Other examples of web services used in drug design are the Chemspider chemoinformatic tools [13] or the Open PHACTS platform [14]. Alternatively, as shown also in Figure 1, modelers can login into the VM and access eTOXlab directly, using its command line or graphic user interface (GUI) for implementing and validating new models or maintaining existing ones.

The most basic VM used for eTOXlab distribution contains only open source modeling tools: RDKit [15] for chemistry manipulation, PaDel [16] for computing molecular descriptors, Standardizer [17] for structure normalization, and R [18] for model building and validation. eTOXlab incorporates an in-house developed, highly efficient, implementation of PLS, and our novel ADAN method [19] for assessing the applicability domain and providing reliability indexes for the predictions. However, this VM is only a basic configuration and modelers can install additional software tools. This design has the advantage of imposing no limits to the type of software that can be installed. The basic platform includes no software that could prevent its use due to intellectual property issues but it is possible to install commercial software in versions customized for users that own the required licenses. Also, eTOXlab imposes no restrictions with respect to the web service application programming interface (API) implemented in the VM. Standard eTOX VMs implement an API developed ad-hoc for the project (Sanz F et al.: Integrative modeling strategies for predicting drug toxicities at the eTOX project, forthcoming). The demo eTOXlab VM (freely downloadable from http://phi.imim.es/envoy/) implements a very simple API described in the Additional file 1: Annex IV. Others APIs (e.g. OpenTox [6]) could be eventually supported.

Regarding the software itself, eTOXlab is a collection of object-oriented Python modules. The main class *model* implements methods representing the main tasks involved in the building, validation and use of QSAR models, as it is shown in Table 1. The methods of the class model implement source code that carries out the corresponding tasks making calls to other, internally implemented, methods or calling external software installed in the VM. Table 1 details the interfaces to external tools pre-installed in eTOXlab. These include many commercial tools for which there are no appropriate open source options, like Moka [20,21] for adjusting the ionization of the compounds according to a given pH or CORINA [22,23] for converting 2D structure to 3D. Deriving interfaces for other tools is simple and can be done with little effort.

**Table 1:** **Description of the main methods of** ***model*** **class**

**Method of the** ***model*** **class**	**Task description**	**External software typically called by the class**
*normalize*	Structural standardization	Standardizer [17]
	Change ionization status (to a given pH)	RDKit [15]
	2D to 3D transform	*Moka [20,21]
	Check if the compound was present in the training series	*CORINA [22,23]
*extract*	Compute molecular descriptors	PaDel [16]
	Extract biological properties from the input structures	*Pentacle [24,25]
		*ADRIANA Code [26]
*build*	Build a predictive model	R [18]
	Validate the model evaluating it goodness-of-fit and predictive quality (by cross-validation)	
*predict*	Produce a predicted value for the biological property of interest	R [18]
	Asses the reliability of the prediction	

Software marked with an asterisk is not open source and would require a software license.

eTOXlab contains a highly efficient implementation of multivariate methods like principal component analysis (PCA) and partial least squares (PLS). PLS can be applied as PLS regression (PLS-R) or discriminant analysis (PLS-DA) depending on the type of variable representing the biological property of the series (quantitative or qualitative, respectively). In the latter case, the system incorporates an automatic cutoff estimation for obtaining the best balance between specificity and sensitivity. The methods also implement leave-one-out (LOO) cross-validation for estimating the predictive ability in either case. The quality of the PLS models can be optimized automatically applying FFD variable selection [27].

Using eTOXlab with default setting makes possible to build a QSAR model in few minutes. Simple settings, like the choice of molecular descriptors (MD), the type of molecular structure standardization and the machine learning used can be easily defined by editing a single file (Additional file 1: Annexes I and II for an step-by-step example). However, the real power of eTOXlab resides in the possibility of overriding the original methods of the *model* class, replacing them by “child” methods that can be customized without limits, while inheriting all the capabilities of their parents. This means that developers can re-implement their own methods or making calls to the software of their choice and still the model will retain all the native common services (e.g. version management, web services, applicability domain testing, etc.…) present in the parent class. An example of how overriding methods can be applied to customize eTOXlab models can be found in the Additional file 1: Annex III. The model customization possibilities are described in more detail in the Model building subsection.

**Figure 2:**
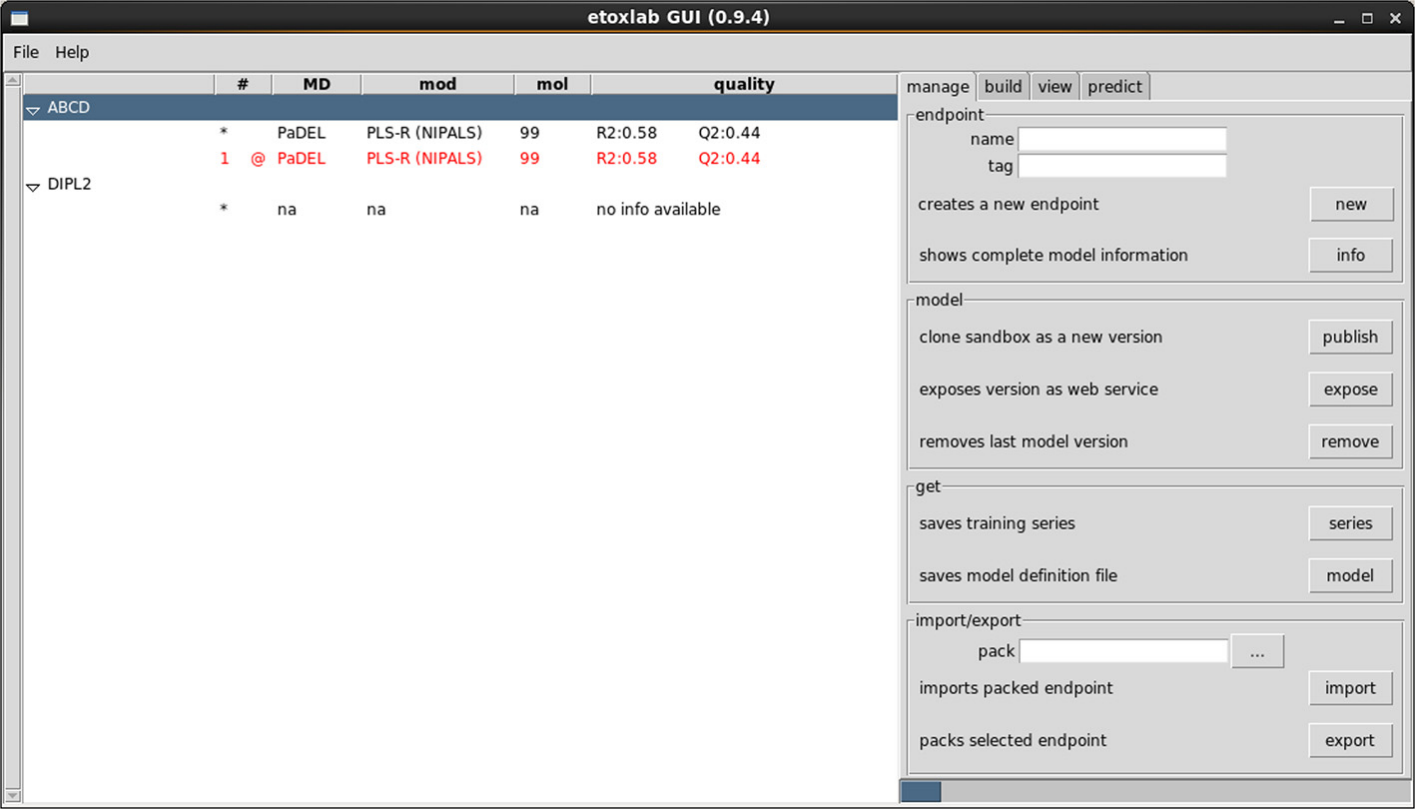
**Screenshot of the eTOXlab graphic user interface.**

In line with the architecture shown in Figure 1 eTOXlab can be used in two different ways. Model developers can login in the VM and use a command line interface or the graphic user interface (Figure 2) for building the models, testing the prediction and performing model maintenance. End users interested only in obtaining predictions have no need to login in the VM, they can consume exposed models through a web service from any computer able to connect to the VM. In the project eTOX, a devoted centralized server (eTOXsys) gives access all the models using a sophisticated API that supports asynchronous jobs (Sanz F et al.: Integrative modeling strategies for predicting drug toxicities at the eTOX project, forthcoming). The demo VM implements a much simpler API and a basic web interface (Additional file 1: Annex IV).

**Figure 3:**
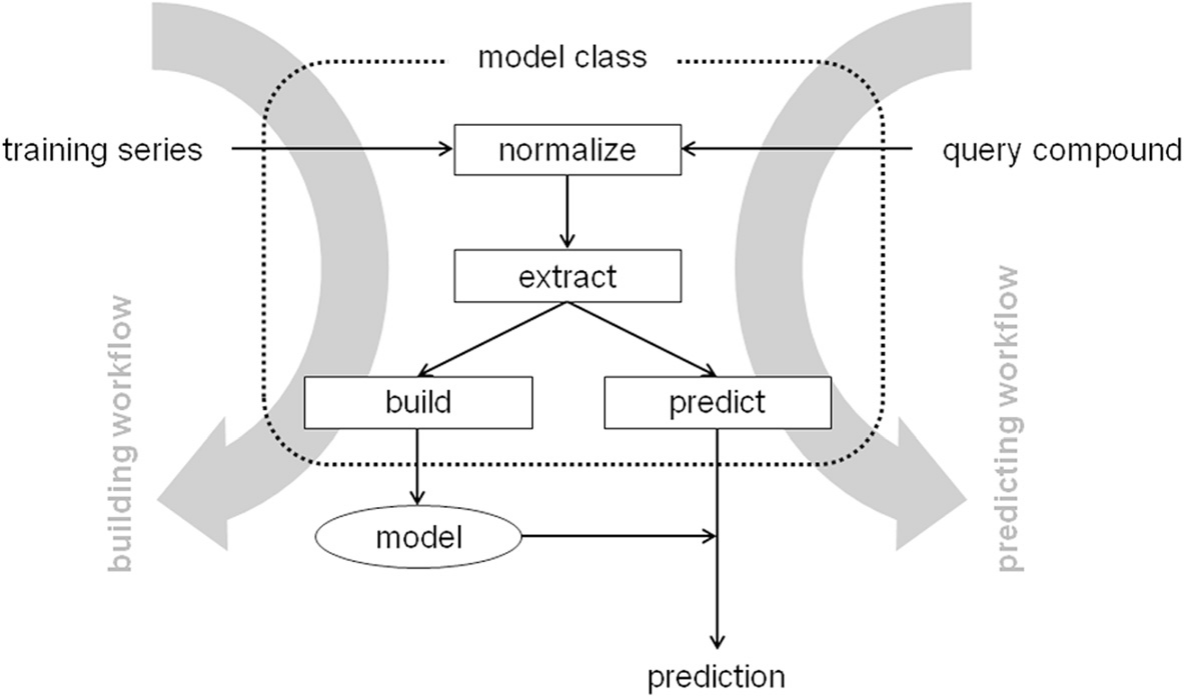
**Schema of the main eTOXlab workflows: prediction (right hand side) and building (left hand side), indicating the input and output, and the** ***model*** **methods called.** Notice that both workflows use the same *normalize* and *extract* methods.

eTOXlab implements building and prediction workflows that define the order and the calls to be made to the methods of the *model* class (Table 1), as represented in Figure 3. For building a model, the first step of the building workflow consist in normalizing the structures of the training series (*normalize* method), these structures are then used to compute the numerical description of their structures and to obtain the biological annotations present in the original file (*extract* method). With these, a QSAR model is built and validated (*build* method). The net result of this process is a predictive model, which is stored internally at the server. For carrying out predictions, the first step of the prediction workflow is to normalize the input structure and to compute the molecular descriptors exactly as it was done for the compounds of the training series. In eTOXlab this requirement is guaranteed because the very same methods (*normalize* and *extract)* are applied. The molecular descriptors together with the stored models are then used for producing the prediction (*predict* method). These pre-defined building and prediction workflows, like any other method of the *model* class, can be also overridden allowing the user to implement models that use completely different workflows.

## Results

### eTOXlab functionality

Details about the software functionality will be presented in this section by describing how it can be applied at the different stages of the QSAR life cycle: model building, predicting and model maintenance. At the end of this section we present an interesting application of the prediction system for generating models with confidential structures that can be exported and shared without risk of disclosing sensitive information. It must be noted that eTOXlab provides a dual interface for model developers and maintainers: all functions can be accessed using either a command line interface or a GUI.

### Model building

In eTOXlab, models are built with a single call to the *build.py* module. The *build.py* module can generate a new model with default settings simply by entering the name of the file containing the training series. The following call builds the model: “build -e NEW -v 0 f training.sdf”, where NEW is the tag that identifies the model, 0 is the version number and training.sdf is a SDFile containing the structure of the training series and their experimental biological activities (e.g. pK_i_ or pIC_50_) in an internal field. By default, eTOXlab assumes that the name of this field is “activity”, but it can be easily changed, as described in the Additional file 1: Annexes I and II. Alternatively, the models can be built using the GUI, by defining the same information. So far, eTOXlab only admits SDFiles as a valid format for the training series. This choice can be justified by the widespread use of SDFiles in the drug development field and the possibility to embed additional data (e.g. the biological properties) but also other data which can be used to generate the predictions. We do not discard supporting other molecular formats in future versions.

The VMs can store an unlimited number of models, each one located in a separate file system tree and identified by a unique tag. Every model version is assigned a tree branch with its own instance of the parent model class (so called *imodel*) defined in a separate copy of the *imodel.py* file. As mentioned before, the new model class inherits all the built-in methods and capabilities of the parent class and it can be customized by the user. Version folders contain their own copy of the training series and of other files storing the model results (not directly accessible to the end-users), thus constituting a completely self-contained model package.

Calling the module *build.py* runs the workflow shown in Figure 3, using the methods represented therein. By default, the model is built using open source tools. The structure normalization uses Standardizer, for standardizing the 2D structures of the training series. 2D PaDel were used as molecular descriptors and PLS as machine learning method. At the model development stage, all models are assigned version *0*. Once the model is built, relevant model quality parameters are shown. For quantitative variables and PLS regression eTOXlab presents r^2^ values for Goodness-of-fit, leave-one-out cross-validated q^2^ and standard deviation of error of predictions SDEP, for every model dimensionality computed. Scatter plots of experimental vs recalculated and predicted values are also generated and, in the case of the GUI, presented in the screen. If required, the user can adjust the *imodel* settings and repeat the model building. Once the user is satisfied, the version can be published using the *manage* command. The model is assigned a sequential number and is ready to be exposed as a web service accessible from outside the VM. Multiple versions of the same models can be implemented and all of them remain accessible for prediction.

Models can be customized adjusting diverse configuration settings. For example, the user can configure the software that generates the molecular descriptors and define the variables to use (e.g. selecting 2D or 3D PaDel descriptors) or configure the machine learning method (e.g. define the scaling of the molecular descriptors or the number of latent variables to use in the PLS models). In some cases, information embedded within the SDFile can be used to complement the computed molecular descriptors, thus adding extra flexibility to the methods. Also, eTOXlab implements natively a FFD variable selection algorithm [27] which can be switched on for optimizing automatically the predictive quality of PLS models. All these configurations settings can be changed by editing the *imodel.py* file, as described in Additional file 1: Annexes I and II.

As mentioned, more complex models can be implemented by overriding original methods of the *model* class with new methods in the *imodel* child class. This approach takes advantage of the object oriented programming capabilities of Python for providing full flexibility: methods can be customized with no limitations, while untouched methods keep providing the full features of the parent *model* class and a seamless integration in the predictive environment. The eTOXlab distribution accessible at http://phi.imim.es/envoy includes examples and templates for using modeling tools not installed in the basic VM. Also, the Additional file 1: Annex II shows a simple example of how the prediction methods can be overridden for implementing a decision-tree model.

### Predicting

At the production stage, most predictions request will be handled by the exposed web service. This will accept as input the structure of a query compound in a SDFile format and run the prediction workflow shown in Figure 3. As previously discussed, the sharing of common methods in the building and prediction workflows guarantees that the query structure will be submitted to exactly the same protocol used for the training series and the maximum consistency of the results. During the model development stages, the command *predict.py* or the predict tab of the GUI (Figure 2) can be used to run this workflow and simulate the results provided by any model version. One of the tunable settings of *imodel* activates the detection of query compounds already present in the training series. When this option is on, the model presents as the predicted value the experimental value already determined for this compound. An important feature of eTOXlab is the calculation of reliability indexes for every prediction. By default, the system implements the ADAN method [19], which was developed ad hoc as a robust reliability index/applicability domain assessment method for the eTOX project. However, as for any other eTOXlab feature, this method can be replaced by other alternatives.

Predictions can be generated using the GUI. In this case, the results are shown in a separate window (Additional file 1: Annex II) and exported to CVS format or as SDFile with the predicted values, the applicability domain and the 95% CI inserted inside.

### Model maintenance

Models might need to be updated for several reasons; better software components became available, model developers decide to introduce changes in the workflow or get access to new compounds that can enrich the training series. This latter reason is very frequent and typically requires a systematic, periodic model updating. In any case, the goal of model maintenance is to increase the overall quality of the predictions and expand the model applicability domain.

In eTOXlab, models are built using a single command. In the case of incorporating new compounds this operation is easily performed by concatenating the original training series with the new compounds and building a new model version. In the case of workflow changes, the model maintenance only requires editing the *imodel.py* file, as described in the model customization above. If the model verification confirms that our changes have produced an improvement, the new model can be published as a new version and exposed as the version used for prediction by the web services. It must be stressed, however, that previous versions are still present and usable. This is particularly useful in forensic investigations, where the source of historic decisions must be traced back to the data that originate them. The whole procedure for model updating can be run periodically without human intervention using appropriate scripts.

In addition, eTOXlab provides basic version management tools allowing listing all available versions (including the date of creation, model details and quality), removing and even exporting them. All these operations can also be carried out using the provided GUI (see Figure 2).

### Model storage and documentation

A common practical problem in model development is to maintain a centralized repository of models, appropriately documented. The system presented here permits to encapsulate models and to maintain a consistent repository of models, including multiple model versions, which can be linked to a centralized database with complete documentation about the model endpoints, characteristics of the training series and model quality. This approach has been implemented in the eTOX project, where every model has been extensively documented according to the project standards, and this information is accessible, with different level of detail, to the end user from the same interface used for submitting the predictions (eTOXsys) (Sanz F et al.: Integrative modeling strategies for predicting drug toxicities at the eTOX project, forthcoming).

### Confidential mode

VM/eTOXlab systems can be seen as portable, self-contained predictive engines. They are ideal tools for academy-industry collaboration, since the predictive systems can be built and trained by academic experts and deployed as readily usable VM to corporate environments. Precisely the decoupling between model-training and model-prediction allows another, very interesting exploitation. Very often, the industry owns large collections of confidential compounds that would be ideal for the development of predictive models. Understandably, the risk of disclosing these structures or the associated experimental information make impossible to share this valuable information, preventing its use in precompetitive collaboration exercises with other companies or with the academia. Solutions proposed for solving this issue (structure masking, use of surrogate information) [28-30] failed to provide a satisfactory answer to the problem.

In this scenario, the VM/eTOXlab represents an alternative strategy. The whole predictive system can be installed behind the corporate firewalls for training models with confidential structures. By using a special model building mode (so called “confidential mode”) the resulting model is produced without retaining any trace of the original training series; once the model is trained, the only information which is stored is the array of PLS coefficients, representing the correlation of every MD variable with the modeled endpoint. Please note that, irrespectively of the training series size, only one value per MD variable is stored. Therefore, this information cannot be used to trace back the values of the MD for the training series, in the same sense that the value of an average cannot be used to trace back the original values used to calculate it. It must be noted that this procedure relies in the removal of the information and not in encryption, hashing or masking of the structure or the MD. No data about the training series is retained and for this reason, the model is suitable for being exported out of the company without compromising any structural information. Furthermore, for maximum transparency, the models generated by eTOXlab using this mode are stored in text format, which can be easily audited and inspected for guaranteeing that no information about the training series is retained in the exported models. In the GUI, models generated in confidential models are clearly highlighted in the model list, to facilitate their identification.

## Discussion

The software presented therein has been in use within the eTOX consortium for nearly a year, supporting the predictive models of the main prediction system (eTOXsys) (Sanz F et al.: Integrative modeling strategies for predicting drug toxicities at the eTOX project, forthcoming). The models implemented so far were very diverse. In some cases, the software has been heavily customized for supporting non-QSAR models, which range from simple decision trees to extremely complex models combining molecular dynamics with linear interaction energy (LIE) methods [31]. In a collaborative project like this, the use of a single modeling framework simplifies greatly the implementation of a common interface (API) between the diverse models and the prediction interface. Also, the use of a common VM template permits distributed development practices: models are developed and tested at the modeler sites and the final components are easily deployed and re-assembled at the end user facilities.

By design, eTOXlab is not closed software, but a collection of Python modules that can be easily customized to suit the needs of highly heterogeneous modeling methodologies. eTOXlab can be seen as a wrapper that provides the modelers all the services required for integrating their models in a complete prediction system providing well structured model building and prediction workflows, structure normalization and version management. This means that the modeler can focus its efforts on implementing and refining its model within the VM template, with the confidence that this model will be immediately available for use trough the web services and will integrate seamlessly with the rest of the prediction components.

The working cycle implemented in eTOXlab is well adapted to the characteristics of the diverse users of predictive systems in drug research working environments. Final users of the models can obtain predictions using a web based GUI to access the web services and therefore, no contact with the software is required. Model developers typically have good computing skills and, in our experience, were able to implement their models within eTOXlab with very little effort. Indeed, this system allows completely freedom to install in the VM any required software and to link eTOXlab with pre-existing code. Finally, computational chemist in charge of the model maintenance could carry out routine operations (like model retraining) in a very simple way, because the model building workflows were an integral part of the model design.

All in all, the software provides a very convenient solution to the main issues pointed out at the introduction. Thanks to the implementation as a VM it is easy to deploy and to install. The VM can be executed by diverse virtual machine players and is not hardware demanding while the prediction systems are offered as web services that can be easily accessed using any web browser. eTOXlab, unlike other modeling software, provides specific support for all the steps of the QSAR model life cycle: building, validation, prediction and re-training, as it was shown in the Results section. This system guarantees full consistency in the handling (structure normalization and molecular descriptor computation) of the training series and the predicted compounds. Model maintenance is straightforward and existing models can be easily re-trained with new data and all the model versions remain accessible. Furthermore, eTOXlab integrates ADAN, a modern and robust method for the quantifying the reliability of the predictions and assessing the applicability domain of the model.

At present, eTOXlab is in active development. Machine learning algorithms embedded in the software are being improved, adding more cross-validation methods and improving their efficiency. The interface with existing tools (like R) is being improved for making it more intuitive. Also, latest versions incorporate new visualization tools integrated into the GUI. More importantly, eTOXlab is being currently applied in our group for the development of sophisticated models, mainly in the areas of drug safety. Apart from publishing the results, we plan to share the resulting models in eTOXlab packed format, thus allowing other interested scientists to make use of them locally, on their own compounds.

## Conclusions

We have presented here eTOXlab, modeling framework supporting the whole life cycle of predictive models in industrial environments. The technologies used by this software (VM, web services, object-orient programming) as well as the software design itself, provide simple, efficient and elegant solutions to the main practical problems involved in the use of predictive models in production settings. Unlike other solutions, based on generic workflow tools, eTOXlab has been specifically designed for this purpose and has been tested in eTOX, an international consortium of 11 academic and 19 industrial partners. eTOXlab is at the core of the eTOX predictive system (eTOXsys) and has already been tested in both academic and industrial environments.

The system described here constitutes also an example of self-contained, portable, prediction engine. As such, it is an ideal tool for supporting collaboration between industry and academia: models built at academic environment can be easily deployed and installed within the company firewalls for being used as prediction black boxes. The local installation is a good alternative to on-line solutions without the inconvenient of sending sensitive structures over the Internet. The VMs, which were pre-configured with open source tools, can be easily customized by installing any licensed software owned by corporate users.

All in all, the software presented here has the potential of becoming a widely used platform for implementing predictive models in production environments and promoting the collaboration between industry and academia.

## Availability and requirements

**Project name:** eTOXlab

**Project home page:** Source code available at https://github.com/manuelpastor/eTOXlab. A self-contained virtual machine can be downloaded from http://phi.imim.es/envoy/

**Operating system(s):** Platform independent

**Programming language:** Python

**License:** GNU GPL version 3

**Virtual machine requirements:** The virtual machine is provided as a single file of 2.8 Gb in standard OVA format. The recommended configuration of the host server is a CPU supporting hardware-assisted virtualization, 4 CPU cores and 8 Gb RAM. The guest system is preconfigured to use 1 CPU core and 2 Gb RAM. The size of the virtual disks is pre-set to 18 Gb.

**Any restrictions to use by non-academics:** none

## Additional file

Additional file 1:
**Annex I.** Building and using a QSAR model in eTOXlab using the command line interface; Annex II. Building and using a QSAR model in eTOXlab using the GUI interface; Annex III. Example of method overriding in eTOXlab; Annex IV. Demo Application Programming Interface.

## Abbreviations

ADAN: Applicability domain analysis
API: Application programming interface
GUI: Graphic user interface
LIE: Linear interaction energy
LOO: Leave one out
MD: Molecular descriptors
PC: Principal component
PCA: Principal component analysis
PLS: Partial least squares
QSAR: Quantitative structure-activity relationships
SDEP: Standard deviation of error of predictions
VM: Virtual machine
