# Supplementary material for: eTOXlab, an open source modeling framework for implementing predictive models in production environments
Source: J Cheminform. 2015 Mar 11;7:8. doi: 10.1186/s13321-015-0058-6 (PMC4358905; doi:10.1186/s13321-015-0058-6)

Annex I. Building and using a QSAR model in eTOXlab using the command line interface

Here we describe a workflow for building and using a QSAR model in eTOXlab based on a data set of CACO-2 provided by Hou et al. in *J. Chem. Inf. Comput. Sci.*, 2004, 44 (5): 1585–1600 (http://dx.doi.org/10.1021/ci049884m). The aim is to illustrate some eTOXlab functionalities using the command line interface.

First we download and unzip the datasets into the virtual machine.

```
wget  http://pubs.acs.org/doi/suppl/10.1021/ci049884m/suppl_file/ci049884msi20040403_083100.zip
```

```
unzip ci049884msi20040403_083100.zip

Archive:  ci049884msi20040403_083100.zip
  inflating: test_set.sdf
  inflating: training_set.sdf
```

Files training_set.sdf and test_set.sdf are available.

We create a new endpoint called "ABCD" with description "/abcd/1"

```
manage --new -e ABCD -t "/abcd/1"

version created OK
```

In order to define the properties of the model we request the default model with:

```
manage -e ABCD --get=model -v 0

File retrieved OK
```

A file named "imodel.py" is available in the current folder. This file contains the "imodel" class that is a child of the "model" class, so methods defined here will override corresponding method of the parent "model" class. For the CACO2 model we will just edit the "init" method. In particular, we will define that the activity value is coded in the field "caco2" of the input SDFile, no normalization should be performed, PaDel descriptors should be used, and a PLS model with 3LV should be used. The changes are done with the idle editor.

```
idle imodel.py
```

After changing imodel.py the result is:

```
# -*- coding: utf-8 -*-

##    Description    eTOXlab model template
##
##    Authors:       Manuel Pastor (manuel.pastor@upf.edu)
##
##    Copyright 2015 Manuel Pastor
##
##    This file is part of eTOXlab.
##
##    eTOXlab is free software: you can redistribute it and/or modify
##    it under the terms of the GNU General Public License as published by
##    the Free Software Foundation version 3.
##
##    eTOXlab is distributed in the hope that it will be useful,
##    but WITHOUT ANY WARRANTY; without even the implied warranty of
##    MERCHANTABILITY or FITNESS FOR A PARTICULAR PURPOSE.  See the
##    GNU General Public License for more details.
##
##    You should have received a copy of the GNU General Public License
##    along with eTOXlab.  If not, see <http://www.gnu.org/licenses/>.
```

```
from model import model

class imodel(model):
    def __init__ (self, vpath):
        model.__init__(self, vpath)

        ##
        ## General settings
        ##
        self.buildable = True
        self.quantitative = True
        self.confidential = False
        self.identity = False
        self.SDFileName = 'name'
        self.SDFileActivity = 'caco2' ##<= changed


        ##
        ## Normalization settings
        ##
        self.norm = False ##<= changed
        self.normStand = True
        self.normNeutr = True
        self.normNeutrMethod = 'moka'
        self.normNeutr_pH = 7.4
        self.norm3D = False

        ##
        ## Molecular descriptor settings
        ##
        self.MD = 'padel'   ##<= changed          # 'padel'|'pentacle'|'adriana'
        self.padelMD = ['-2d']                    # '-2d'|'-3d'
        self.padelMaxRuntime = None
        self.padelDescriptor = None


        ##
        ## Modeling settings
        ##
        self.model = 'pls'
        self.modelLV = 3    ##<= changed
        self.modelAutoscaling = True
        self.modelCutoff = 'auto'
        self.selVar = False
        #self.selVarMethod = GOLPE
        self.selVarLV = 2
        #self.selVarCV = 'LOO'
        self.selVarRun = 2
        self.selVarMask = None

        ##
        ## View settings
        ##
        self.viewType = 'property'    # 'pca' | 'property' | 'project'
        self.viewBackground = False
        self.viewReferenceEndpoint = None
        self.viewReferenceVersion = 0

        ##
        ## Path to external programs
        ##
        self.mokaPath = '/opt/blabber/blabber110/'
        self.padelPath = '/opt/padel/padel218ws/'
        self.padelURL = 'http://localhost:9000/computedescriptors?params='
        self.pentaclePath = '/opt/pentacle/pentacle106/'
        self.adrianaPath = '/opt/AdrianaCode/AdrianaCode226/'
        self.corinaPath = '/opt/corina/corina24/'
        self.javaPath = '/usr/java/jdk1.7.0_51/'
        self.RPath = '/opt/R/R-3.0.2/'
        self.standardiserPath = '/opt/standardise/standardise20140206/'
```

Now we build the model with the training data set with

```
build -e ABCD -f training_set.sdf -m imodel.py

('training_set.sdf', 77)
Progress: [###################] 100.00% Done...
cross-validating...
LV 1 R2:0.439 Q2:0.291 SDEP:  0.628
LV 2 R2:0.538 Q2:0.305 SDEP:  0.623
LV 3 R2:0.718 Q2:0.218 SDEP:  0.660
Model OK
```

The next step is to use the model to predict the test dataset. The output is the predicted CACO2 value, an applicability domain index and the 95% CI for the predicted value.

```
predict -e ABCD -f test_set.sdf  -v 0

-5.83198 0  1.29419
-6.45577 2  2.58838
-4.05182 4  0.00000
-4.83560 0  1.29419
-5.53917 1  1.29419
-6.73768 2  2.58838
-6.42743 1  1.29419
-4.65175 0  1.29419
-5.06969 0  1.29419
-4.83893 0  1.29419
-6.17705 3  2.58838
-4.23142 0  1.29419
-5.19872 0  1.29419
-4.29936 0  1.29419
-5.09670 1  1.29419
-5.39153 0  1.29419
-5.13877 0  1.29419
-5.96280 2  2.58838
-4.67481 0  1.29419
-6.06141 3  2.58838
-5.58145 0  1.29419
-5.72423 2  2.58838
-4.30183 4  0.00000
```

Once the model is ready to use we can publish it to create a copy of this version on the model repository

```
manage -e ABCD  --publish

/home/modeler/soft/eTOXlab/src/ABCD/version0001
```

Stored versions can be exposed as web services and then be used from outside of the virtual machine. This only requires to type

```
manage -e ABCD -v 1 --expose

version exposed OK
```

Annex II. Building and using a QSAR model in eTOXlab using the GUI interface

Here we describe a workflow for building and using a QSAR model in eTOXlab based on a data set of CACO-2 provided by Hou et al. in *J. Chem. Inf. Comput. Sci.*, 2004, 44 (5): 1585–1600 (http://dx.doi.org/10.1021/ci049884m). The aim is to illustrate some eTOXlab functionalities using the graphic user interface (GUI).

First we download and unzip the datasets into the virtual machine.

```
wget  http://pubs.acs.org/doi/suppl/10.1021/ci049884m/suppl_file/ci049884msi20040403_083100.zip

unzip ci049884msi20040403_083100.zip

Archive:  ci049884msi20040403_083100.zip
  inflating: test_set.sdf
  inflating: training_set.sdf
```

Files training_set.sdf and test_set.sdf are available.

We start the graphical interface by clicking on the "etoxlab" icon in the desktop.

For creating a new endpoint called ABCD with description "/abcd/1" we simply type these values in the "name" and "tag" input fields on the upper right corner and press the "new" button

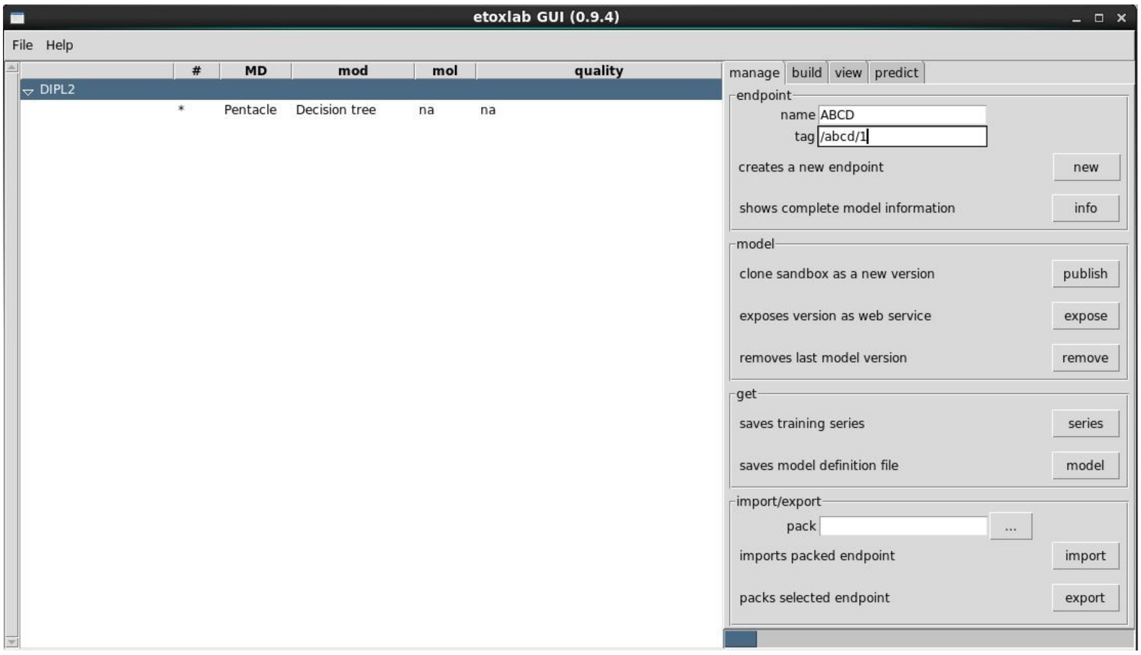

The new endpoint is created and shown in the list of existing endpoints and models

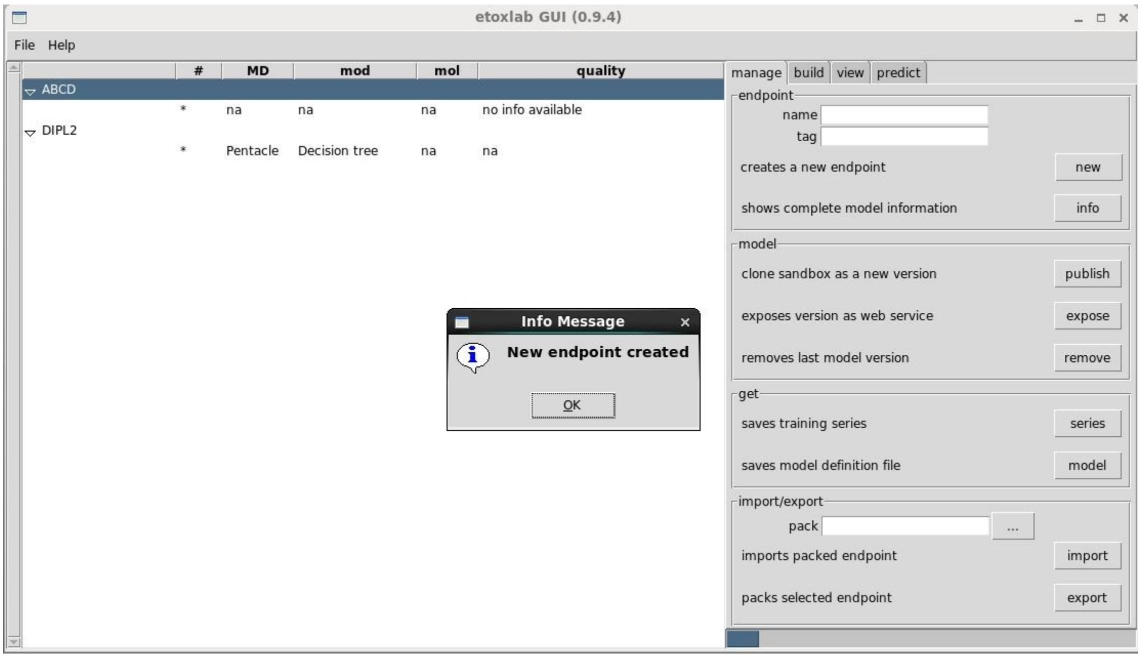

We build the model by selecting the "build" tab. Select the training series "training_set.sdf". Regarding the model, we must configure that the activity value is coded in the field “caco2” of the input SDFile, no normalization should be performed, PaDel descriptors should be used, and a PLS model with 3LV should be used. Pressing the "..." button besides the model will open an idle editor where we can introduce the changes mentioned in Annex I, that basically consist in:

- Setting "self.SDFileActivity" to "caco2"
- Setting "self.norm" to "False"
- Setting "self.MD" to "padel"
- Setting "self.modelLV" to 3

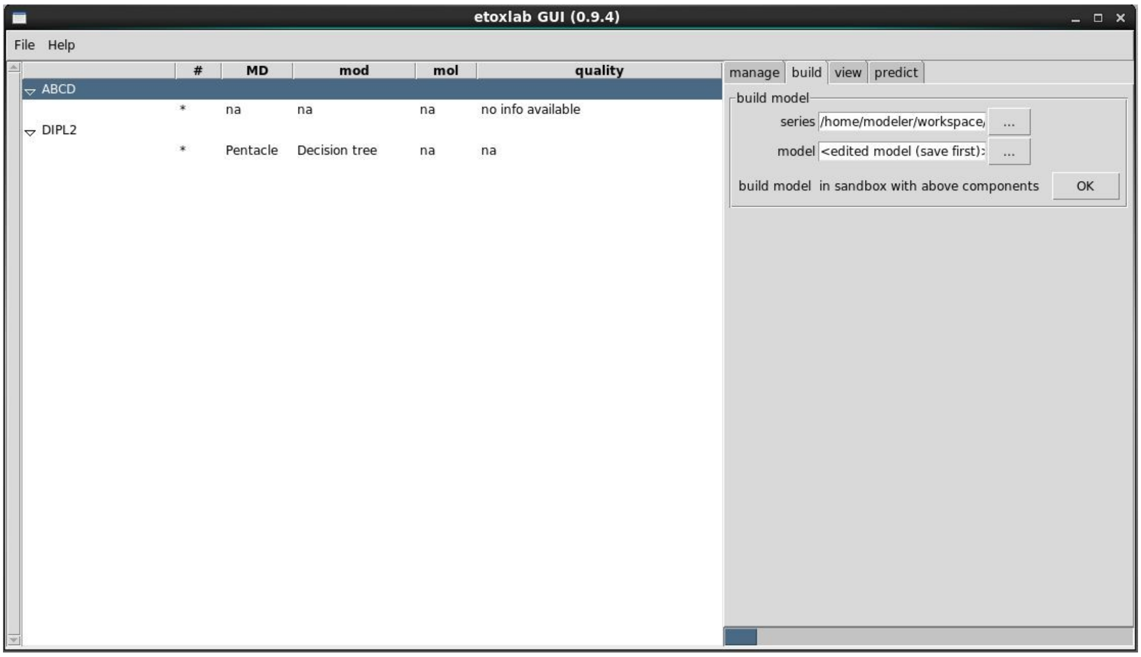

Then we press OK and wait for a few minutes.

When the model building is finished, the values of R^2 and Q^2 are shown in the model tree. Additionally, the GUI generates and displays scatter-plots with the experimental vs recalculated and experimental vs predicted values for all model dimensionalities, which are useful for diagnosing the model quality.

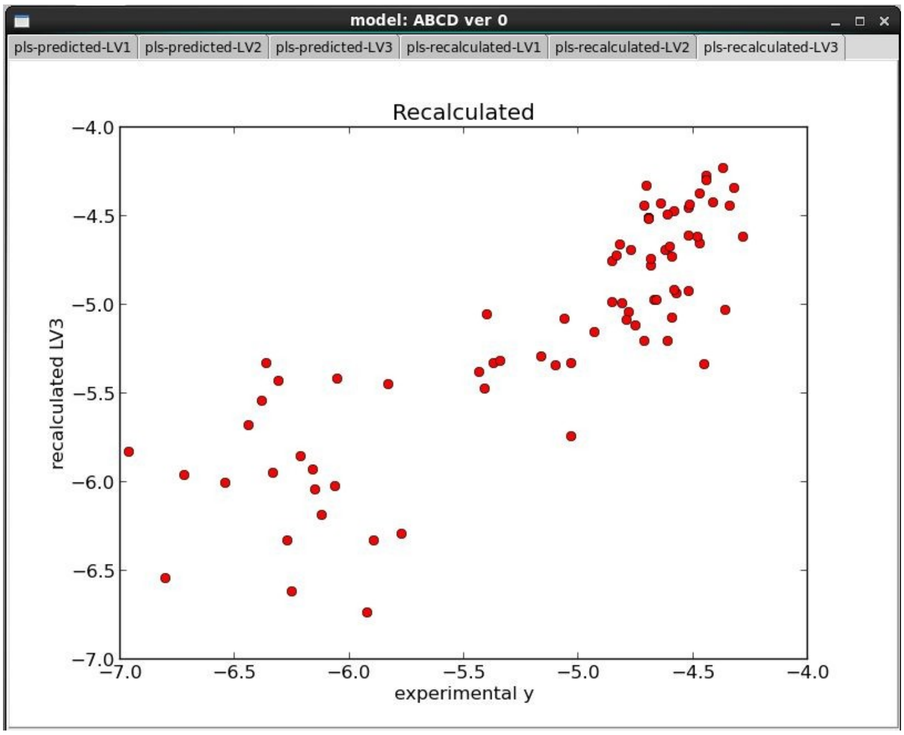

The new model can be used for prediction immediately. Select the "predict" tab, enter the name of the query series and press the OK button.

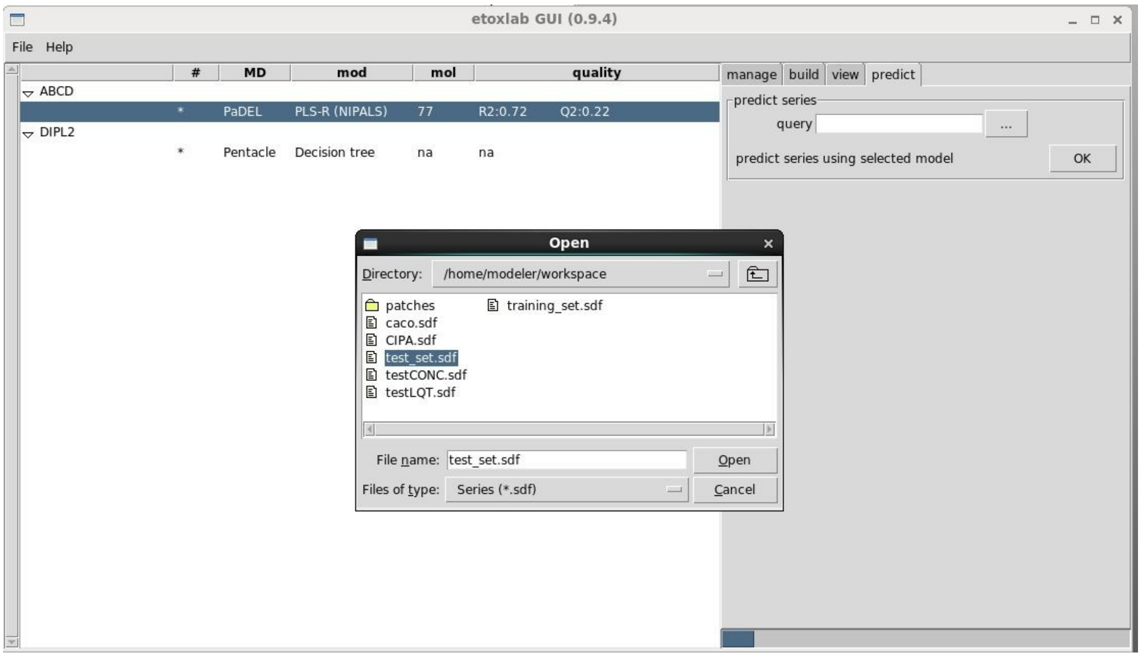

The prediction results are shown in a separate window from where the results can be exported in CSV format or as an annotated SDFile.

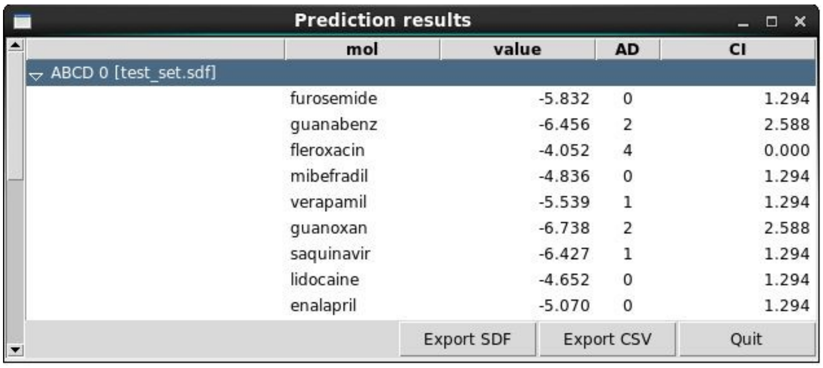

Once the model is ready to use we publish it to create a copy of this version on the model repository. This can be done from the manage tab simply selecting the version and pressing the "publish" button.

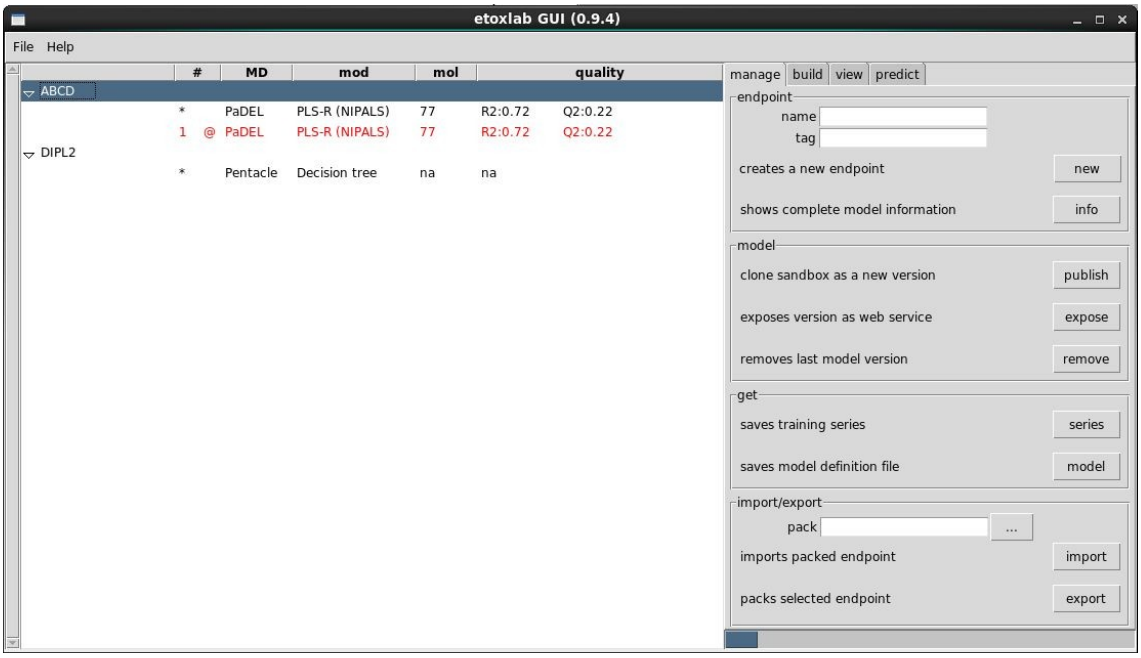

Stored versions can be exposed as web services and used from outside of the virtual machine simply selecting the model version and pressing the "expose" button. Exposed versions are highlighted in red with the "@" symbol in front

Annex III. Example of method overriding in eTOXlab

In order to illustrate the method overriding technique we present here how to implement in eTOXlab a very simple rule-base prediction method. This method was extracted from Tomizawa K. et al. Physicochemical and cell-based approach for early screening of phospholipidosis-inducing potential. *J Toxicol Sci.* 2006, 31 (4):315-24. (http://www.ncbi.nlm.nih.gov/pubmed/17077586)

A rule-based method does not require building a model. Therefore, we only need to override methods of the prediction workflow in the *model* class.

The procedure starts exactly as described in Annex I and II, but the editing of the local imodel.py requires inserting the definition of the new methods, as described below. Please note that in no case we need to edit the original source code, just add the following text at the bottom of the imodel.py file.

We begin by editing the method "predict", which has been simplified to execute only two tasks: call "computeLogP" and use the results to call a new version of "computePrediction".

Then we write the code of "computePrediction". This latter method applies a simplified version of the rules described in the original article; if the compound is neutral or negatively charged, it is considered phospholipidosis negative. Compounds with charge +2 or compound with charge +1 and logP higher than 1.61 are considered phospholipidosis positive. Compounds with formal charges higher than +2 are considered out of the prediction range.

```
def computePrediction (self, logP, charge):
    result = 'negative'

    if charge == 1:
        if logP[0] >= 1.61 :
            result = 'positive'
    elif charge == 2 :
        result = 'positive'
    elif charge > 2 :
        return (False, 'charge out of range')

    return (True, result)


def predict (self, molFile, molName, molCharge, detail, clean=True):
    # default return values
    molPR=molCI=molAD=(False,0.0)

    success, molMD = computeLogP (molFile)

    if not success: return (molPR,molAD,molCI)

    success, pr  = self.computePrediction (molMD,molCharge)
    molPR = (success, pr)
    if not success: return (molPR,molAD,molCI)

    if clean: removefile (molFile)

    return (molPR,molAD,molCI)
```

There are other two methods that must be overridden in this example: "setSeries" and "log". These simply avoid storing information about the training series (non-existing in this case) and provide information about the methods used to generate the prediction ("Decision tree").

```
    def setSeries (self, molecules, numMol):
        self.infoSeries = []


    def log (self):
        self.infoModel = []
        self.infoModel.append( ('model','Decision tree') )

        result = model.log(self)
        return (result)
```

Once the editing has been completed, the model is ready for testing. In this particular case, there is no need to build the model and it can be used for prediction directly.

Annex IV. Demo Application Programming Interface

In the demo VM, the web service is accessible at port 9001. From inside, it can be called by any browser calling to http://localhost:9001, followed by a valid URL

URL	HTTP verb	Input data	return data	HTTP status codes
/info	GET		application/json: info_message response	200
/available_services	GET		application/json: available_services response	200
/predictform	GET		text/html: web form	200
/calculate	POST	multipart/form-data encoding:<br>- model tag<br>- SDFile	application/json: calculate_call response	200<br>500 (in case of malformed POST message)

/info
Returns basic info about the provider of the models

Example of "info_message response" schema:

```
{"provider": "FIMIM",
"homepage": "http://phi.imim.es",
"admin": "Manuel Pastor",
"admin-email": "manuel.pastor@upf.edu" }
```

/available_services
Returns the list of all available prediction services.
The predictions field in will contain an array of the tags of the models available to make predictions.

Example of "available_services response" schema:

```
{"predictions" : ["ABCD"] }
```

/predictform
Shows a form allowing the user to make predictions. The user is asked to select the model to use and to upload the SDFile with the 2D structures of the query compounds

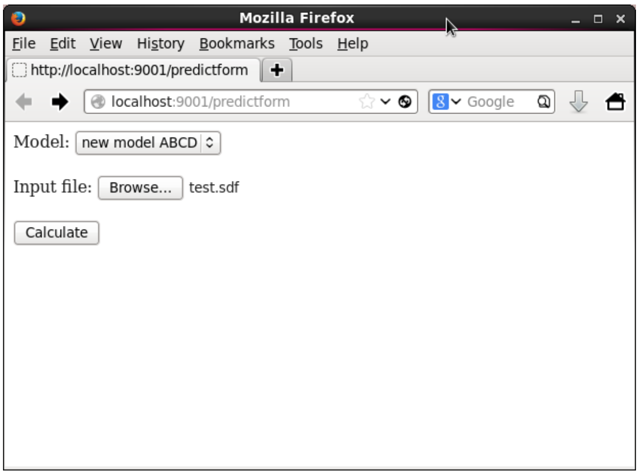

/calculate

Returns the prediction for a model and a SDFile provided by the user.
The model is specified by the tag provided by the "/available_services" call.
The model tag and the SDFile are encoded as multipart/form-data.
To encode the tag we use the “model” field and to encode the SDFile the “uploadfile” field.

Example of "calculate call response" schema:


```
[
        {"cmp_id": "0",
         "success": "True",
         "value": "-6.0743503303",
         "AD":   {
                "success": "True",
                "value": "0",
                "message": ""
                },
         "RI": {
                "success": "True",
                "value": "1.14595589767",
                "message": ""
                }
        },
        {"cmp_id": "1",
         "success": "True",
         "value": "-5.33778477437",
         "AD":   {
                "success": "True",
                "value": "1",
                "message": ""
                },
         "RI":   {
                "success": "True",
                "value": "1.14595589767",
                "message": ""
                }
        }
]
```


Therefore, for every compound we obtain:

- "compound_id": index of the compound in the SDFile.
- "value": the value of the prediction
- "AD": applicability domain of the prediction (in ADAN method format, see manuscript)
- "RI": reliability index of the prediction (95%CI)

For every "value", "AD" and "RI" there is a "success" value that indicate if the computation was correct ("True") or not ("False")

The aspect of the output in the web interface is as follows:

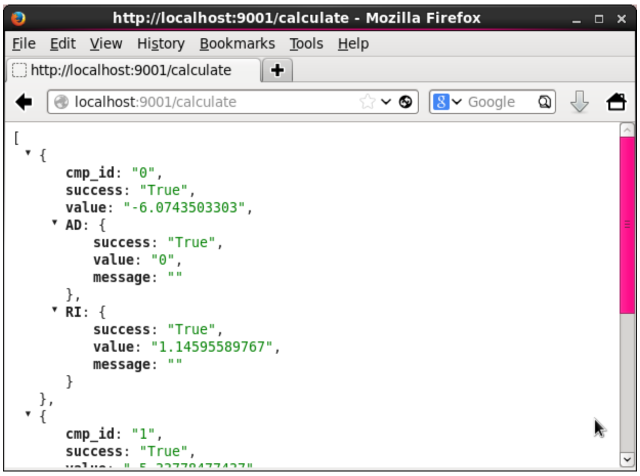

Supplement: Additional file 1: — Annex I. Building and using a QSAR model in eTOXlab using the command line interface; Annex II. Building and using a QSAR model in eTOXlab using the GUI interface; Annex III. Example of method overriding in eTOXlab; Annex IV. Demo Application Programming Interface. [file 13321_2015_58_MOESM1_ESM.pdf]
